# Essays on Medical Education and the Medical Profession in the United States

**Published:** 1832

**Authors:** Daniel Drake


### WESTERN JOURNAL

OF THE

MEDICAL AND PHYSICAL SCIENCES.

JANUARY, FEBRUARY AND MARCH, 1832.

### Essays and Cases.

**Art. I.**—*Essays on Medical Education, and the Medical Profession in the United States.*—By Daniel Drake, M. D.

### ESSAY V.

Causes of Error in the Medical and Physical Sciences.—— Legislative Enactments.*

The causes which prevent the discovery of Truth in the Medical and Physical Sciences, are too numerous to be embraced in a single essay; while many of them are too much concealed to be brought to light, by any but a man of genius and extensive observation. Those of which I propose to treat, are common, and therefore, susceptible of being exposed.

The first of these is the disproportion between the strength and grasp of our intellectual faculties, and the

* This Essay, with those formerly published upon the same subject, in this Journal, has since been published in a separate form; but as the edition is small, and the circulation consequently limited, we presume it will be considered a favor by the majority of our readers, to have the series completed in the Journal.

NUMBER AND VARIETY OF OBJECTS UPON WHICH THEY ARE TO BE EXERCISED.

In styling himself "lord of the creation," man has, perhaps, consulted his pride more than his powers. Certain it is, that he is not less dependent on the other objects of creation, than they are on him. These objects, either animate or inanimate, are, to him, absolutely innumerable—they touch him on every side, and stretch from him indefinitely in all directions. Their relations with him and with each other, are never the same for two successive moments; for the group which *now* surrounds him, will soon surround him *no more;* and while he yet contemplates it, changes of composition and character have taken place, which he could neither forsee nor prevent. Even individual objects confound him; and when actually in the focus of his physical or intellectual vision, undergo transformations, which surpass his comprehension, and teach him lessons of wisdom and humility. Suppose that all his observations were correct—would this enable him to arrive at philosophical truth? It would not, unless those observations were extended to all the individual qualities and relations of every object requiring examination. In constructing a system of science, it is not merely necessary to have no *bad* materials, but an adequate number of *good* ones. *Ignorance* is said to be better than *error*; but this adage refers chiefly to the condition of the mind, in regard to further improvement; for, in establishing a system of science, ignorance and error are perhaps, equally unpropitious.

Nature, moreover, is not only too *unlimited* in the *number* of her objects, but their structure and functions are too complicated to be studied without mistake. The very "clods of the valley" presents to the philosopher, in their composition, a problem which he cannot undertake to solve without error,—the grass, which he tramples under foot, when he stoops to examine it, offers appearances which he can neither understand nor report with accuracy—while his own body—an assemblage of intricate organs, mysteriously united, and harmoniously acting and re-acting on each other, from a principle of motion equally mysterious, presents him with a case, in which the utmost exertion of his powers of observation and reflection will not secure him from false conclusions. If such be the inefficiency of genius, how deplorable must be the failure of imbecility:—let dulness, then, inquire with anxiety, and publish in doubt and hesitation.

ANOTHER REASON WHY WE SLOWLY ARRIVE AT PHILOSOPHICAL TRUTH, IS THAT WE SEEK IT WITHOUT DUE PREPARATION.

The chemist who attempts the analysis of compounds, without having previously studied the laws of affinity, prosecutes and ends his labors in error; while the pharmacopolist who alters the formulæ and recipes of the profession, without a previous knowledge of practical chemistry, decomposes and adulterates what he seeks to improve. In the science of Materia Medica, the man who expects to advance our therapeutick knowledge, without a previous acquaintance with the nature and chemical history of medicines, and their *modus operandi* on the body in health, falls into an error almost as great, as if he were to rely exclusively on that *preliminary* knowledge, to direct him as to their *curative* powers, which can only be known by the additional labour of administering them to the sick, and registering their effects. The operative surgeon who omits the cultivation of surgical anatomy, necessarily commits ravages upon those whom he undertakes and anxiously desires to relieve. Finally, the physician who aims at a knowledge of pathology, without first devoting himself to the study of the animal economy, and frames his rules of practice in his own office, instead of the chambers of the sick, may labour with diligence, but will never command success. His conclusions, although not always wrong, cannot often be correct. His prescriptions are like gilded pills of noxious qualities, in which the poison is concealed from the eye, but works desolation and death in the unfortunate patient.

In all these cases, and many others that might be cited, the cause of error, is a violation of the rule of philosophising which commands us to proceed from the "known to the unknown."—It is not a deficiency of object, of time, of means or of intellect, that precludes the attainment of truth, but a neglect of due preparation, a presumptuous disregard of things that lie near, for those which are more remote; but which, when examined, cannot be understood, because they have been prematurely reached.

The next cause of failure in the pursuit of philosophical truth, is more deeply rooted. It consists not so much in the deficiencies, as the errors of early education.

From the very nature of our associations in infancy and childhood, much of what we learn is erroneous; and throughout the whole period of our studies, except when engaged in the exact sciences, we daily imbibe falsehood with truth; and, unconscious of the combination, retain the whole under the name of knowledge. The tenacity with which the mind clings to these false impressions, is often not less than the facility with which, in early life, it receives them; and as they exclude an equal portion of real knowledge, and vitiate all our conclusions, their influence cannot be too strongly deprecated. It becomes both pupils and physicians, frequently to review their past studies, and anxiously to inquire into the circumstances under which their knowledge was acquired. By such retrospection, carefully and humbly made, they will often be able to detect the occasion, or the *dictum*, which led them into error, and thus purify and fit their minds for the reception of new truths.

Another impediment to the acquisition of truth, is fickleness of attention.

Childhood is the epoch of mutability, in which impressions rapidly succeed and supersede each other. Children do not dwell long enough on any object, or group of objects, to acquire correct and ascertained ideas of their properties and relations, and hence they investigate badly. This propensity to pass from subject to subject, is a principle of our nature; and is designed to introduce us to a first interview with the multitude of surrounding objects, with which it is the business of after life to become more intimately acquainted. But like every other principle of action, it requires control and discipline. Appertaining, as we have seen, especially to childhood and youth, it belongs to a good education, to keep it subordinate in manhood. Those in whom this is neglected, grow up deficient in habits of protracted investigation, and seldom dwell long enough on any subject, to comprehend it fully. Such persons may be styled audlt children, and the number is not a few. Gazing for a moment on the superfices of many objects, but looking into the deep structure of none, they seldom perceive the truth, as in most cases, it lies far beneath the surface.

ANOTHER CAUSE OF ERROR CONSISTS IN FORMING CONCLUSIONS, WHILE WE SHOULD STILL BE OBSERVING.

Persons of this intellectual temperament may see deeply, but they look in one direction only; and of course, form an opinion, before all the facts in relation to the case, have been reviewed. They might be compared to the judge, who decides on the testimony of one of the parties, unconscious that another waits for an audience. The judgment may be honest, and a correct inference from the facts before him, but still it is wrong. Many of the errors which vitiate the experimental sciences, as well as the affairs of social life, are referable to this head; and as the authors of them are conscious, not only of loving truth, but think they have investigated their premises warily, it is not uncommon to see them cling with pertinacity to their conclusions. A singularity exhibited by such persons is, that at different times, they are the honest advocates of opposite opinions. The facts, which at one period were presented to them, and led to a particular inference, are forgotten; and, subsequently, those of an adverse tendency are casually offered to their notice, when with equal honesty and promptness, they stand forth as the champions of a contrary doctrine. The fault of this character, consists in imperfect and limited suggestion—

a sort of intellectual *strabismus* or squinting—by which the field of mental vision is narrowed, and the partial mistaken for the universal. The opinions of such men are entitled to respect for their sincerity, but not to adoption for their soundness. They may be true, but should always be presumed erroneous. The proper remedy is a constant recurrence to protracted and comprehensive research; that all the facts in the case may be fully represented in the deduction—when this is realized, the judgment is true, and then only.

THE NEXT OBSTACLE TO THE DISCOVERY OF TRUTH, IS VIVIDNESS OF FANCY.

Many cases of madness have seemed to grow out of this exuberance. It tends to abstract us from external objects, and causes inattention to the impressions which they make, or might make upon us. Such philosophical fanatics, require but a glimpse, to afford them, as they believe, an accurate knowledge of the properties and relations of the most complicated group of objects. Their senses do not resemble a camera obscura so much as a magic lanthern. A single sensation can spring a long train of ideas; and cause new creations to succeed each other, like the spectres in that machine, and generally with as little conformity to truth and nature. Philosophers of this excessive mobility, are right only by accident, and it is equally accidental, if they discover themselves to be wrong. They do not so often change from error to truth, as from one illusion to another. They suppose themselves men of genius, when, in fact, they are endowed with but one of its attributes. They are great builders of hypothesis, but all their pyramids are inverted. They never investigate closely, but to sustain a pre-conceived notion, and then instinctively reject every fact which militates against it. Their ardor and obstinacy are often so great, as to inspire confidence in their opinions, and make proselytes of those who should labour to expel their delusion, and bring them back to the sober realities of the external world. Such an enterprise, would, however, in most cases, prove ineffectual; and hence students and young physicians should guard vigilantly against the earliest illusions of this *ignis fatuus* of the mind.—If it once attract them from the straight and narrow path of inductive philosophy, their faculties will speedily glide into the mazes of error; and, if practitioners, their prescriptions will become sentences of death, instead of warrants of health and life.

The next obstacle to the discovery of truth, is impatience.

Patience, which is not a greater virtue in morals than in science, has been brought into disrepute, chiefly by the sect of philosophers which I have just described; if they indeed, can be called a sect, who unfortunately are never embodied, but contaminate all others. It is difficult to bring patience into favor and fashion. Being very commonly an attribute of inquisitive dullness, many young men of lively feelings, have not courage to practice it, lest they should be suspected of belonging to that class, in which no human being ever yet enrolled himself without a struggle. The highest eulogy that could be pronounced upon patience in research, is that it constitutes the great secret of that success, which men of inferior talent so often attain. It *must*, therefore, be a powerful auxiliary, and why should any man reject its assistance? To say that strength of intellect can render it unnecessary, is not true, unless that intellect be employed upon inferior objects; and genius should condescend to dispute with stupidity, for the subjects which legitimately belong to the latter. In his own elevated sphere, the ablest philosopher will find patience essential to the development of truth. Nature is not an oracle of Delphi, to be bribed or flattered into responses. Her severe majesty requires that she should be interrogated patiently, and listened to with meekness; and even when thus besought, her replies may not always be as prompt and explicit as we could desire; but they will neither be interested nor erroneous.

At the risk of being found tedious, I must appropriate some additional paragraphs to this part of our subject. There are students and physicians, intrinsically industrious and patient in the exercise of their *mental* powers, who, nevertheless, have a fastidiousness in regard to manual labour, especially what is unpleasant, which materially interferes with the discovery of truth, in their own and its auxiliary sciences. Let us pause for a moment, and, notwithstanding the enumerations in our previous essays, inquire what they are: Anatomy and Physiology, Sugery, Clinical Medicine and Materia Medica, Chemistry and Pharmacy, Zoology, Botany and Mineralogy, Physical Geography, Geology, Pneumatics, Hydrostatics, Meteorology, Astronomy, Mechanics, and the Chemical, Mechanical, and Fine Arts, are, in fact, but a part of them. Physical science is, indeed, the science of material nature, and comprehends as its own proper objects, not only the globe, and every thing living and dead, within its bowels or spread over its surface, with all which constitute or inhabit its atmosphere; but also, the boundless system of celestial orbs, which, as suns, and planets, and satellites, and comets, enliven the dark and dreary regions of infinite space. Now, let us place before ourselves, an imaginary picture of the objects to which I have referred, and then contemplate their number, varieties and analogies, their relative positions, their individual properties, their varied uses, their attractions and repulsions, their dependencies on each other, and the influence which man exerts on them, and they on him, and we shall be prepared to estimate the value of our remaining attainments, if an acquaintance with the forms and qualities of matter were expunged from our minds. But this would be impossible, for material nature is the foundation of all our knowledge, and in proportion as we neglect its study, science becomes uncertain and unsubstantial—speculation supersedes experiment—hypothesis replaces theory—and syllogisms usurp the office of induction.

Our proper business, then, is the study of material objects, and these must be examined by our organs of sense, to be rightly understood. If this examination be reluctant and superficial, the results will of course be imperfect. If we come to it in a temper of fastidiousness, and shrink from the necessary exertion, because we may have been unused to manual labor, or some parts of that which should be done are unpleasant or repulsive, we shall seldom grasp the truth. If we suppose, that not having been compelled to labor for subsistence, but, nursed in the lap of wealth and luxury, we may leave the drudgery of science to hands already soiled in the field, or hardened in the work shop, we shall, at last, be disappointed in our dearest hopes; and mortified to see those whom habits of toil and application, have prepared to vanquish every difficulty, stretch immeasurably beyond us, in the race of emulation and professional fame. In the pursuit of physical truth, an examination of the objects of nature, is the first and greatest work; and he who brings to it the best manual dexterity, the greatest self-dependence, and most untiring industry, with the fullest command of his feelings, in relation to scenes and objects, which are often offensive and sometimes loathsome, will, *cœteris paribus*, be most successful in his researches. It is a prevalent and pernicious error, that the labors of the devotee of physical science, are chiefly intellectual; or that we may view nature from select positions, and acquire all the necessary knowledge, by perspective views. A much closer intimacy is requisite to a full acquaintance. If we read the biography of the greatest men, who have adorned the profession, or dignified the ranks of physical science, we shall find them almost as remarkable for their labours of body as of mind. The former, directed to the objects of their ambition, supplied the *pabulum*, by which the latter was cherished. Such is the order of nature, and no student or physician, who prizes truth or reputation, should ever depart from it.

PRIDE IS A STUBBORN OBSTACLE TO THE DISCOVERY AND RECEPTION OF TRUTH.

Meekness of spirit, is scarcely a more necessary predisposition for the reception of divine, than scientific truth. The selfsufficient philosopher never enquires into his defects. When he contemplates himself, it is rather to admire his excellences, than to correct his imperfections. He views the former with a mote, the latter with a beam in his 'mind's eye.' He employs a mirror, which reflects only the beauties of his character; and should it, at any time, become true to nature, he turns from the unwelcome glimpse of his deformities, as from things to be concealed and forgotten, not corrected. To expose them to society would mortify him still more. He is even reluctant to display his improvements, as they would imply previous defects of knowledge, and approximate him to mortals of a common mould. A student of this class often prefers to remain ignorant, rather than appear so; but it is, especially in advanced life, that pride interferes most fatally with improvement in science. In youth, vanity sometimes tempers this indomitable passion; but in process of time, it not unfrequently subdues every opposing sentiment, and, becoming fortified by habit, governs the character of the individual, with the spirit of a tyrant. Hence the obstinate perseverance in ancient errors, the cynical sneering at late discoveries, the dogmatism, the pomp and the real or affected scepticism, which in medicine, so often render old age ridiculous, and sometimes seriously impede the dissemination of new truths. I know of no cure for this *fungus hœmatodes* of the mind, when it has become deeply rooted, but in youth it may be eradicated, and every student should seek to cast it out.

Excessive deference for authority—in some degree the opposite of pride—contributes equally to the perpetuation of error.

An overweening regard for authority in the sciences, is the offspring, either of a slender understanding or a timid spirit, still further enfeebled by bad education. It shows itself, not merely in an unsuspicious assent to alleged facts—a pardonable credulity—but in an implicit adoption of the conclusions of eminent men, when we should examine for ourselves, both their premises and reasonings. The latter species of intellectual servility has done much harm to the profession, and through it to society at large. In general, we are under the necessity of receiving as true, that which the archives of medicine present to us as fact; for it is impossible to repeat every experiment, and many observations can never be made a second time, because the same combination of circumstances may not recur; but nothing should be taken on trust, when it can be avoided; as that which is reported correctly, may have been seen incorrectly, and the professions of truth with which a subject is introduced, may be designed by the author, to protect it from suspicion. But the *reasonings* of an author, a professor or a colleague, are legitimate subjects of scrutiny, and he who passes timidly over them, admits an inferiority, which fearless investigation might convince him did not exist. He becomes the slave of opinions, instead of the servant of truth; and contributes not more to the diffusion of falsehood, than to the degradation of his own character. As the opinions of great men have the widest circulation, and receive the most implicit adoption, it becomes all who can be incited to the duty of examining what they are about to receive, to subject those of eminent writers, to a carefuller criticism than is necessary for the speculations of authors of lower powers, and more limited fame. If this had been always done, the genius which has been devoted to the profession, must have carried it far beyond its present elevation.

A DEFECTIVE LOVE OF TRUTH, IS ANOTHER CAUSE OF ERROR IN THE PROFESSION.

The love of truth, although an original principle of human nature, has various degrees of native energy; and from the operation of several causes, may decline, till we come to look upon error with complacency, and at last not only tolerate, but publish falsehood. Very few principles of our nature, are, indeed, beset with so many causes of corruption. I will name but two—self-interest and self-love. The combined influence of these, on the love and practice of truth, in morals and society at large, is seen and admitted by all; and it is equally great in the medical sciences, where, although less deprecated, it is perhaps still more mischievous. Self-interest and self-love, it is true, sometimes animate us in the pursuit of truth; but it is only when reward awaits its attainment, or disadvantage or disgrace would flow from failing to reach it. Unhappily for the interests and dignity of human nature, such cases are few in number; and hence these restless and unwearied principles of selfishness, are more seldom enlisted in the service of truth than falsehood. While students, it is uncomfortable to feel, and sometimes mortifying to acknowledge, that we do not fully comprehend a subject; and, strange as it may seem, we, now and then so far compromise with the love of truth, as to pretend, and, perhaps, sometimes almost believe, that we are less ignorant than is the fact. After having passed through our pupilage, and taken rank with the initiated, it becomes our business to interpret nature to those below us; and as we should then consider it still more discreditable to be at any time found wanting, the motive to practice imposition is strengthened.—But it is as physicians and surgeons, when we expect to receive patronage, less in the proportion of our true knowledge than of our plausibility, that the temptation to deceive, both ourselves and others, acquires an irresistable and frightful energy. As the naturalists find it necessary to supply from analogy, the lost parts of a Mammoth skeleton, before it can satisfy vulgar curiosity, so physicians feel the importance of presenting an aspect of perfect knowledge, when they would acquire public confidence. This imposture, not often detected, is still more seldom frowned upon by the hoodwinked community, whose unreasonable demands have invited it; and, as impunity promotes the repetition of crime, one imposition follows another, till the love of truth falls gradually into ruins; and the heart once honest and open, becomes at last polluted with the principles of a criminal selfishness. The guileless lineaments of unsophisticated nature, are now exchanged for the studied imitations of art; and the question no longer is, what does truth require, but what will best administer to the insatiable cravings of self-interest, and where lie the moral limits which cannot be overstepped, without injury of reputation?—Mark the physician who has thus de-generated, and you will no longer perceive in him any sacred veneration for professional truth. If he purchase books, it is rather to *appear* learned, than to become so; and when he reads, it is more to extend his notoriety than his knowledge. He may not purposely avoid the truth; but he no more trims his mid-night lamp, and diligently compares the recorded observations of others, with his own, made through the day; he relaxes in his efforts, to note down the facts which fall ander his observa-tion: or, what is more criminal, suppresses or perverts them, to suit his prejudices, or display his conduct and character in a favorable aspect. In his practice, he often omits the perform-ance of duties, because they may be servile and troublesome; when, from such omission, the truth could not be ascertained, nor his patient treated with success. In cases of extreme dan-ger, he will neglect the investigations that are indispensable to a true knowledge of the case, and occupy himself on the means of satisfying those interested in the result, that he has done all that could be done, if not all that was required. He magnifies the danger through which those have passed, who appeared to be seriously ill when they were not; and expects thus to bal-ance the account of profit and loss, when a patient dies whom he ought to have saved. In the application of remedies, he yields to prejudices and apprehensions, which it is his duty to encounter, and, if possible, overcome; and neglects the use of means which are necessary to the safety of his patient, lest cen-sure should follow their unsuccessful employment. When re-quired to confer with another physician, he looks more to the preservation or extension of his fame, than the life of the patient; now doubly jeopardized, by the complaint and the consultation. If the attending physician, he makes false or imperfect state-ments—adding or subtracting, in his narrative of the past treat-ment, such items as he thinks necessary to a favorable estimate of his sagacity;—if the consulting physician, to acquire a name for liberality, he sanctions with criminal courtesy whatever has been done, and recommends its continuance; or rejecting the whole, aims, like a cannibal, to fatten his popularity on the despoiled reputation of an able, but artless associate. Finally, should he at any time attempt to lay before the profession the results of his experience, he writes as certain artists paint, merely for effect;—looking to an increase of his reputation, more than the dissemination of truth. Or, captivated by the prospect of temporary applause, rather than the desire for permanent fame, his histories are not made correct transcripts, even of his experience; but resemble caricatures, which strike the vulgar by the disproportion of their parts, or the unnatural depth of their light and shade. Were they equally harmless, it would be happy for mankind, but unfortunately, they are poison cast into the fountain, and before its waters can purify themselves, hundreds may drink and die.

A DEFECTIVE AMBITION, IS THE LAST SOURCE OF ERROR TO WHICH I SHALL REFER.

The want of ambition, contributes signally to retard the progress of medical science. A majority of those who belong to the profession, are destitute of the excitement which it imparts; and, consequently, deficient in those exertions by which only the profession can be advanced. In the selection of boys for the study of medicine, no reference is, in general, made, either to their talents or aspirations; and most of them grow up and go down to the grave, inemulous of distinction, and blind to every recompense, which cannot be recorded on their ledgers. The circumstances of our country, have a large share in the production of mere routine practitioners. In the midst of the general diffusion of political and religious knowledge, literature and science are but little cultivated or respected, except in the larger towns and about our seminaries of learning; and hence the acquirements of medical gentlemen are not always properly appreciated. Emulation, moreover, requires contiguity and contact; and is most energetic, where the pressure of rivalship is greatest. But in the United States, from the sparseness of population, and the small size of most of the towns and villages, physicians are too detached and scattered, to excite each other's ambition, or to concur in operations for the improvement of the profession. Thus, many whose ambition, under a different state of things, might have prompted them to great and successful efforts, while away their whole lives, without having the dormant fires of their ambition once kindled into flame. In the cultivation and practice of a science which is imperfect and speculative, no principle of action is so powerful as ambition. The love of money will animate physicians in the acquisition of business, but not to the discovery of scientific truth. The pursuit of the latter, often, indeed, interferes materially with an accumulation of the former—which, with a few exceptions, has flown into the hands of those who have neglected the cultivation of the profession. The love of knowledge is a nobler affection, but cannot be relied upon to promote the discovery of truth, or extend the boundaries of our science. Physicians of this class, delight in truth and detest error; but having more of contemplation than action, spend their lives in *acquiring* knowledge, and neglect its appropriation. They are misers who hoard *fine* gold only; but depend exclusively, on an increase of their stores, for the pleasure which should flow more copiously from expenditure than accumulation. They industriously gather what they are, in general, too indolent to apply, or even bequeath; living as it were, for themselves only; and manifesting a character of refined and unoffending selfishness, which we can neither blame nor applaud. The body, the labors and the fame of such an one perish in the same grave; and the history of his virtues; his taste and genius—his attainments, purified in the furnace of a noble understanding—his unextinguishable love of truth—and his day dreams of patriotism—are written bnt on the narrow tablet of the grave, and read by those only, who visit the mansions of the dead. I would not envy such an one, the gratification which he experiences, in his inemulous retirement from the theatre of ambition and usefulness; nor can I agree that the love of posthumous reputation, should be classed among the vanities of human life. The lover of truth, who is content with its acquisition, has less enjoyment, than he who superadds useful application to accurate attainment; and of all the original principles of our nature, the desire of honorable fame, is not only most beneficial to the world, but one of the most ennobling to the soul which it animates.

We must not confound *this* ambition, either with pride or vanity. The former preserves us from little actions, but never impells us to great—the latter contents itself with vulgar admiration, and incites us to those performances, only which bring immediate applause. Pride looks to society for no reward, either present or prospective: vanity undertakes nothing on trust—is not willing to rely on posterity for payment—exacts daily returns from the surrounding multitude, and slakes its thirst with draughts of flattery and adulation. Ambition, in itself, has no element either of good or bad. It works as a mere animating principle; and rouses us to deeds of desperation and death, or schemes of beneficence and life, according to the moral sense and the objects of each individual. When it subjugates the heart, and arrays itself in the armor of misanthropy, it delights in nothing, but new advances over a terrified and prostrate world; but subordinate to the heart, it becomes the bright sun of society, and cheers and vivifies whatever its beams may fall upon. Such is the ambition, which should glow in the bosom of every physician; he would then see no mountain too lofty to be scaled, nor so overhung with murky vapours, that he could not discern the clear and beautiful sky that stretches out beyond its summits. Warmed by love for mankind, enamoured of his profession, and animated by the hope of rising higher, than all who had trodden its rugged steeps before him, he would move on his upward march, with a never tiring step. Not too proud to employ every honorable means, however humble; nor so vain as to loiter, by the way, to catch the syren sounds of popular applause, he would see and think and dream of nothing, but the grandeur which surrounds the objects that allured him on. He would suppose all the time, that these being attained, he might sit down in the midst of them, and give himself up to enjoyment. But the man of true ambition, never stops to revel in the camp of victory. He delights in action and anticipation, and looks with rapture from his new elevation, at the distant and loftier pinnacles, which till then were hidden from his view. Finally the physician of honorable ambition, has no quiet sleep while any are before him. He would not keep them back, but advance himself—he does not regard them with envy, but emulation. He even extends to them his *respect*, but this requires no ordinary effort, and the magnanimity of his character, is seen in the candor, with which he can bring himself to view their merits, and identify their fame with the glory of his profession.

I shall conclude this declamation on some of the causes which oppose the progress of medical science, in the United States, with a passing remark on two or three points that merit animadversion.

1. It deserves to be considered, how far the union of the practice of Physic, Surgery and Obstetricks, in the same individual, has contributed, in the United States, to affect the progress of the profession.

It is a plausible opinion, that these branches should be united, in the same individual, as they are manifestly connected in theory. Nearly the same principles of Physiology and Pathology are common to the whole, thongh they differ in Nosology and Therapeuticks. No one can be prepared to practice either, without a full elementary acquaintance with the whole. But when we come to the business details of the profession, who is capable of so executing them—still cultivating its general principles—as to become great in all ? Certainly none, but men of genius and intense industry. All others sink into mere routinism. From an attention, too incessant and diversified, to the details, they neglect the philosophy of the profession; until, at last, they can give but few reasons for their many prescriptions. It will not be denied, that such practitioners may do a great deal of good, in the midst of some mischief; but they are not the men, who advance the science. They are mere operatives, and work on the plans of others. They are unacquainted with the *defecti et desiderata* of the profession. They have not time, nor, after a few years, ability to study their cases, in connexion with its principles; but rely exclusively on their own experience, and the recipes of their brethren. They never originate schemes of reform or improvement, project original experiments, or develope new elements of knowledge. All this results from too much variety in their practical operations; for which the appropriate remedy, would be concentration upon some part of the profession. To have depth, the diffused waters must be gathered into a narrower channel. The man, possessing even ordinary talents, who devotes himself to Surgery, Medicine, or Obstetricks, instead of the whole, may carry into his practical operations, an accurate and comprehensive knowledge of principles; thus making his experience available, to the rectification of the errors which vitiate them—augmenting their limits, and defining their uncertain landmarks.

On the whole, I am disposed to conclude, that the want of this concentration, is a cause that has retarded, and continues to retard, the progress of the American profession, in scientific improvement; though unquestionably, its members are eminently practical, and generally efficient in their efforts.

2. Among physicians, surgeons, and obstetricians, there are two classes, who run into opposite extremes. They might be termed the philosophical, and practical or empyrical. The former delight in general principles, and with difficulty concentrate their attention upon the practical maxims of the profession: the latter, find such principles above their reach, or deny their correctness, and occupy themselves, exclusively, on the receipts and dogmas of practice. Each sect is right as far as it goes; but each is imperfect. The philosophy of the science is found in its general principles; but we cannot practice correctly without rules, and principles are but the theorems by which rules are formed. We must understand the former to be medical philosophers, and act by latter to be clinical practitioners. With principles, only, we are diffuse and ineffective—with rules alone, either timid to a fault and *suffer* our patient to die, or rash and reckless, and destroy many who might have lived, had death fought single handed against them.

3. The number who venture on the practice of physic, is indefinitely greater, than those who profess to practice surgery in its higher branches. This arises, in part, from the greater number of clinical than surgical cases; but a very different cause, likewise contributes to the disparity. The operations of surgery, expose the ignorance of the practitioner; and, when unskilfully performed, injure his reputation and subject him to the penalties of the law. But in the practice of physic, the detection of errors, by the people, is generally impracticable. Hence many will confess their ignorance of the former, while all are ready to profess a competent knowledge of the latter; although, in reality, physic requires in the aggregate, more profound and varied attainments than surgery. How important it is, then, that the physician should be a conscientious man, who has other motives for investigating the principles of the profession, than the fear of having his ignorance detected and exposed! He should shrink with horror from the idea of prescribing on false premises, or by loose and unphilosophical analogies. He should recollect, that human life is at stake; that a human being, in extremity, is confided to his skill and honor, and that an ignorant or presumptuous stroke of his pen may translate that confiding fellow creature—perhaps his bosom friend—from time to eternity, to confront him with damning testimony on the day of final retribution. Although such practice is not legally felonious, it deserves unqualified denunciation; for in a moral view, where lies the line of distinction, between criminal prescriptions, and those which prove fatal, through the criminal ignorance of their authors? I say *criminal* ignorance, for although, in the abstract, there is no criminality, in being unacquainted with the truth, yet, in the practice of medicine, to be ignorant of facts and principles, which lie within the grasp of common minds under common opportunities, and still to undertake that, which they, only, who are acquainted with those facts can accomplish, is to forget the divine maxim of doing to others as we would be done unto; and, literally to wage war upon human life; not in malice prepense towards any individual, but with the senseless and fatal impartiality of an epidemic disease.

### Essay VI.

No one can dispute the propriety of placing Medical schools under the supervision of the law; and rendering them amenable to the sovereign power of the state. It will avail but little, however, that they are thus authorized, if that power does not govern them with liberality, vigilance, and wisdom. They should be regarded, not as private but public incorporations; designed for the benefit of society, rather than of the individuals who compose their faculties. A medical professorship, is indeed a public office, and should be filled or made vacant from no other motive than the general good. No claims for admission, but those founded on talents and general fitness, should ever be allowed; and the discovery of a failure in these, and this only, should be regarded as a justifiable ground of dismissal. The principle of rotation in office, should never operate to effect the removal of a competent professor.

When a State is about to institute a medical college, and subsequently, it should have a special regard to several points.

1. The location. The proper site for such a school, is a populous city. Uncommon talents in the professors, may secure, for a time, the prosperity of a school in *any* situation; but, misplaced, it is liable, like a plant in barren soil, to perish or decay, when committed to unskilful or indolent keeping.

2. The endowment. Some kind of revenue or an actual appropriation of adequate amount, should always accompany the charter; not as a *bonus* to professors, but to afford them the means of research and illustration.

3. A hospital. This should be as ample as possible, and so planned and regulated, as to be made a clinical school, and a school of morbid anatomy. The rights and duties of the professors and pupils, should be clearly specified; and both made so comprehensive, as to secure for the sick, the best possible attendance; while they, in turn, should repay society and the profession, for the charity they receive, by the benefits which their cases can be made to confer on the pupils.

4. The supplies for the anatomical hall. Exhumation should be prohibited, under the severest penalties, rigorously enforced; but all who were found dead in the streets or fields, or who die in hospitals, poor houses, houses of correction, jails and penitentiaries, and are not claimed by friends, and taken away, to be interred at their own expense, should be delivered to the professors of the college. This would abolish the practice of disinterment, which, otherwise, *must* and *will* continue, until other sources of supply are opened. The opposing demands and denunciations of society, on this subject, indicate that the present is, indeed, but the age of transition from a lower to a higher grade of civilization. Society, in former times, did not require anatomical knowledge, in those to whom it intrusted the limbs and lives of its members: at a future period, it will afford to its medical guardians, the means of acquiring the knowledge it demands.

5. The Trustees. These should be chosen for their qualifications. They should not only be men of talents, intelligence and literature; but from taste, inclined to devote themselves to a faithful execution of their stewardship. Above all, they should be men of honor and impartiality.—tolerating in their hearts neither prejudice nor prepossession—and conducting the institution with a sole view to the public good.

6. The accountability of the trustees to those from whom they derive their authority. This should be made as rigorous as possible. The trust being strictly eleemosynary, should be confided to no board of trustees any longer, than they are found to render it beneficial to those for whom it was granted. Annual detailed reports should be required from the Board, accompanied by reports from the Faculty, and these should be submitted to the scrutiny of select and competent committees, with instructions to investigate closely, and report impartially, on the state of the institution; all which should finally be published, together with the new acts of fostering or corrective legislation, which they may have suggested. By these means, abuses would be detected, and a powerful motive infused into the trustees, to act with the skill, moderation, and justice, which befit those who should be accountable to public opinion, as well as the laws.

Thus founded and governed Medical Schools, are a blessing to mankind, and reflect the highest honor on the governments from which they emanate.

A state which establishes and conducts a medical institution on these principles, will have little need, of what are styled laws to regulate the practice of physic. It will regulate and depurate itself. If the fountain of supply be pure, the stream will seldom be tainted, and its waters will require but little clarification. Should they burst forth in a turbid condition, the work of purification will be found both expensive and difficult. More than half the states of the Union, have laws to regulate the practice of medicine; but I am by no means convinced, that they have ever done any real good to the profession or society. New-York and Ohio have such laws: Virginia and Kentucky none. It remains to be shown whether the profession in the two former, is more respectable than in the latter. I am disposed to believe it is not. Laws which admit to the practice of medicine, those who have not graduated, give many young men a passport to the confidence of the public, who do not deserve that confidence; and could not easily have acquired it without a license. Those, moreover, who are rejected by boards of censors are, in most cases, sustained by society on that very account; and the profession is charged with sinister views. The rejection of a candidate for a degree, by the Faculty of a college, brings him into contempt; but the refusal to grant a license, secures for the applicant a portion of public sympathy and patronage. The reason is not obscure. When *all* the physicians of a state are incorporated, and boards of examiners are appointed by ballot, a great number, inevitably, belong to the corporation, and too often to the examining tribunals, whom the people do not regard as qualified; and they are therefore, not inclined to look quietly on the rejection of a young man, not inferior, perhaps, to those who have put a veto on his aspirations. It is difficult, indeed, for these bodies to establish an authority, that society or even the profession, will recognize; and equally difficult, for them to maintain internal harmony, or adhere to a standard of uniformity in their decisions. The office of censor is essentially one of rotation, and generally, becomes an object of ambition. Its acquisition is often attended with circumstances of management, that are discreditable to the profession; and its administration, still oftener, perhaps, so conducted, as to offend the feelings of those, who have an immediate interest, in the admission or rejection of such candidates, as are their own pupils. Thus feuds are generated, and a whole district degraded, by reciprocal charges of malfeasance in office; which, whether true or false, disturb the harmony of a profession, whose very efforts to lie still, keep it forever agitated. At last, what is a license but a certificate of inferiority ? A licentiate may be a good physician, and become a great man, but still there is an original technical difference between him and a graduate, which every body recognizes; and, as far as testimonials are concerned, he is one, who has not made the attainments, which entitle him to a doctorate. Too many, however, who are thus admitted, and, legally, become members of the profession, rely on the hope, that the mode of their introduction, will be at length forgotten; and never afterwards, seek to qualify themselves for those university honors, to which otherwise they would have aspired.

On the whole, I am convinced, that a state should acknowledge none as members of the profession, who have not graduated; unless it could not have a medical school, and was so remote and insulated, as not to receive an adequate supply of educated physicians. To establish a school, and then to admit into the profession, by legal enactments, those who have not availed themselves of its advantages, is inconsistent, and playing at cross purposes.

At the same time, I would not enact penalties against those who choose to practice without a diploma: for such laws are never carried into effect, and, especially, are opposed by the people; who often sustain a man, when thus persecuted, as they call it, who would, otherwise, have made but little progress in society. For all practical purposes, it would be sufficient, not to allow him to appear in courts of justice, for the collection of his professional earnings, on the sole ground, that he could not show a diploma from an authorized university.

The profession of the Apothecary, should receive attention from our legislatures. The practice of keeping and putting up their own medicines, is going rapidly out of fashion, among the physicians of all the cities and larger towns of the Union. In consequence of this, the compounding of prescriptions, is passing from students of medicine, to the clerks and shop keepers of druggists, most of whom, as well as their operatives, are mere merchants, almost entirely ignorant of the branches of science connected with the business which they follow; and equally destitute of classical learning.

They who put up physicians' prescriptions, should be acquainted with Botany, Materia Medica, Chemistry, and Pharmacy, and possess such a knowledge of the Latin language, as would enable them to decypher, with correctness, what is sent to them. If ignorant in these respects, they are, perpetually, liable to make mistakes, which may defeat the objects of the physician, and even destroy the life of the patient.

The law should, indeed, not only require apothecaries, who undertake to compound the recipes of the profession, to be qualified, for that nice and important duty; but, also, to file and preserve the manuscripts sent to them, that a proper responsibility may be established, and the blame of errors, by which the sick may be seriously injured, fixed on those who commit them. Without this skill and precaution, the lives of patients are in perpetual jeopardy.

I shall close, with a reference to the law of patents. The practice of granting patents for nostrums and panaceas, is discreditable to the age. It originated in a period, when medicine was far from its present state of improvement; and the science of legislation, correspondingly, imperfect. The day has arrived, when juster views should prevail. A chemist may, with propriety, receive a patent for the discovery of invention of a new compound, but not for its administration to the sick. He might, for example, prepare sulphate of morphia, from a cheaper article than opium, and by increasing the quantity, diminish the price to the consumers, and should be rewarded by a patent; but if he asked for an exclusive right, to administer some preparation of that medicine in certain diseases, it should not be given; because, such an administration could not benefit the public, the ultimate object of every exclusive grant. It will be acknowledged by the profession, that no article of the Materia Medica is beneficial, or even safe in every case of the disease, for which it is generally used. To be successful it must be adapted to the actual condition of the patient's system, and this can only be known to a competent observer. A patent remedy, is therefore, an absurdity, and too often a slow poison.

It may be said, however, that empyricism would palm its nostrums on the credulous, if they were not patented. This is true; but it is equally true, that patents are most authentic passports to the confidence of the people; which is well understood by those who prepare quack medicines, and constitutes the chief motive for applying to the patent office; although the alledged reason may be, to prevent counterfeits. Hence the law promotes imposition on the ignorant, whom, in fact, it ought to protect. It is, therefore, prejudicial to society, and should be repealed. To this end, our medical colleges and societies, ought to unite in memorializing the general government. A joint and earnest effort, could scarcely fail; and whether successful or unsuccessful, would reflect credit on the American Profession.

